# The “Preparation for Shared Decision-Making” Tool for Women With Advanced Breast Cancer: Qualitative Validation Study

**DOI:** 10.2196/16511

**Published:** 2019-12-20

**Authors:** Domitilla Masi, Amalia Elvira Gomez-Rexrode, Rina Bardin, Joshua Seidman

**Affiliations:** 1 Avalere Health, Center for Healthcare Transformation Washington, DC United States; 2 University of Michigan Medical School Ann Arbor, MI United States

**Keywords:** shared decision making, clinical decision making, patient preferences, cancer, breast cancer, human-centered design, patient care planning

## Abstract

**Background:**

The range of decisions and considerations that women with advanced breast cancer (ABC) face can be overwhelming and difficult to manage. Research shows that most patients prefer a shared decision-making (SDM) approach as it provides them with the opportunity to be actively involved in their treatment decisions. The current engagement of these patients in their clinical decisions is suboptimal. Moreover, implementing SDM into routine clinical care can be challenging as patients may not always feel adequately prepared or may not expect to be involved in the decision-making process.

**Objective:**

Avalere Health developed the *Preparation for Shared Decision-Making* (PFSDM) tool to help patients with ABC feel prepared to communicate with their clinicians and engage in decision making aligned with their preferences. The goal of this study was to validate the tool for its acceptability and usability among this patient population.

**Methods:**

We interviewed a diverse group of women with ABC (N=30). Interviews were audiorecorded, transcribed, and double coded by using NVivo. We assessed 8 themes to understand the acceptability and usability of the tool.

**Results:**

Interviewees expressed that the tool was acceptable for preparing patients for decision making and would be useful for helping patients know what to expect in their care journey. Interviewees also provided useful comments to improve the tool.

**Conclusions:**

This validation study confirms the acceptability and usability of the PFSDM tool for women with ABC. Future research should assess the feasibility of the tool’s implementation in the clinical workflow and its impact on patient outcomes.

## Introduction

### Background

The full range of decisions and considerations that a woman with advanced breast cancer (ABC) may face throughout her care journey can be overwhelming and difficult to manage. The National Comprehensive Cancer Network guidelines include over 45 treatment regimens for the most advanced stage of breast cancer [1]. As ABC treatment decisions are often preference-sensitive (influenced by an individual’s values, goals, and preferences) and involve significant trade-offs [2,3], shared decision making (SDM) is an important component of high-quality ABC care. SDM is a collaborative process that patients and clinicians use to make health care decisions about tests, treatments, and care plans. Informed by both clinical evidence on the risks and benefits associated with the treatment options and a patient’s preferences, values, and goals, SDM is a critical component of patient-centered care [4].

Research shows that most patients prefer an SDM approach as it provides them with the opportunity to be actively involved in their treatment decisions [5,6]. However, the current engagement of patients with cancer in their clinical decisions is suboptimal [7,8]. Moreover, implementing SDM into routine clinical care can be challenging as patients may not always feel adequately prepared or may not expect to be involved in the decision-making process [9]. Implementation of SDM can improve the patients’ emotional well-being, advance the patient’s or caregiver’s involvement in the treatment process, and promote decision satisfaction [10,11]. Research also shows that patients want to discuss the impact that a treatment choice may have on factors such as their ability to work, caregiver’s responsibilities, and the cost of their treatment [12]. Patients also feel that the cost of treatment should be transparent and part of the decision-making conversation with their health care providers. In fact, in a study with 149 patients with advanced cancer, over 30% rated the financial distress of their treatment as more severe than physical, family, and emotional distress [13].

### Objectives

With the aim of addressing these needs, in 2018, Avalere Health, a research and consulting firm dedicated to enhancing health care in the United States, employed a human-centered design process to iteratively develop a tool to support SDM for patients with ABC. First, we developed a draft prototype of the tool based on the background research on the preferences of patients with ABC and the results of a focus group study held with 8 patients with cancer in collaboration with the Cancer Support Community in 2016. Second, in partnership with Cancer*Care*, an organization that provides free support services to patients and caregivers, the study staff held a group interview with 7 women with ABC to identify their decision-making–related needs and to help design sections of the tool. Third, we created a prototype and held additional one-on-one semistructured interviews with a different set of 8 women with ABC, 2 oncologists, and 2 social workers to receive feedback on how to improve the prototype. Finally, the study staff developed the Preparation for Shared Decision-Making (PFSDM) tool based on the feedback received [14], which is intended to help women with ABC feel prepared to communicate with their clinicians and engage in decision making that is aligned with their personal preferences. It is important to note the collaborative efforts in developing certain sections of the PFSDM tool. Specifically, the development of the section, *Questions to Answer for Your Doctor* was led by oncologists in collaboration with Avalere Health and 2 patient advocacy groups [15]. The PFSDM tool includes 4 sections to (1) support patients with ABC understand the phases of their care experience and key decision points, (2) support patients with ABC think through their personal preferences before their visits, (3) elicit patient preferences and share them with the oncologist before their visit, and (4) guide the patient-clinician conversation during the visit.

The goal of this study was to validate the PFSDM tool for its acceptability and usability for patients with ABC.

## Methods

### Participants

A balanced panel of adult women with ABC, defined as stage III and IV, were recruited to participate in the validation study. We included women with stage III and IV breast cancer after considering the range and complexity of treatment-planning decisions that these women should prepare to discuss with their oncologists. A third-party market research firm used 3 recruitment tactics to find participants: social media posts and advertisements on online cancer support groups; referrals from the members of support groups in New York City and Washington, DC; and informational flyers posted in clinics and hospitals with large populations of patients with cancer. The firm provided a toll-free number that potential participants could call to receive more information about the study. To verify the participants’ conditions, the firm required proof through doctors’ notes or documentation of hospital visits. The research team developed an interview screener to ensure only eligible women participated in the study and to recruit a diverse cross-section of patient characteristics across education levels, age, income, and race and ethnicity. All participants provided written consent to participate in the study. Participants unable to communicate or read English, provide consent, or who did not meet the recruitment criteria were excluded from participating in the study. Participants were surveyed before the interviews (Multimedia Appendix 1) and were provided with the PFSDM tool by mail before the interviews. The survey data are available in a separate publication [16]. Interviews lasted for approximately 1 hour, and each participant took 15 min to 30 min to complete the survey. All participants were compensated for their time.

### Study Design

The interview guide was structured using 2 key research questions and the themes and subthemes outlined in the code book (Multimedia Appendix 2). Whenever possible, questions from previously published literature were used [17,18]. Additional questions were added in consultation with oncologists, survey methodologists, and other subject-matter experts. We used the same interview guide for all interviews. To best capture the participants’ perspectives on the PFSDM tool, the interview guide included both open-ended and closed-ended questions.

### Study Procedure

A total of 2 nonclinical, experienced Avalere Health interviewers, who were not involved in the development of the PFSDM tool, conducted the interviews using the semistructured interview guide (Multimedia Appendix 3). Furthermore, 1 additional Avalere Health staff (the notetaker) was present during each interview to help capture notes and contextual factors. Over the course of 30 interviews, 2 notetakers were involved in transcribing separate interviews. Participants were asked about their overall impression of the PFSDM tool and specific questions about each section of the tool. Interviews were audiorecorded, transcribed, and independently coded by the 2 Avalere Health notetakers using the qualitative analysis software, NVivo 11 Plus (QSR International Pty Ltd, version 11, 2015). The interview data were not anonymized before coding, but patients were only identified by their first names. The research team developed and iteratively updated a code book, which guided the coding process. Overall, 8 themes were assessed to understand the acceptability and usability of the PFSDM tool: (1) understandability, (2) clarity of information, (3) amount of information, (4) suitability for decision making, (5) usefulness, (6) relevance of information, (7) value, and (8) formatting. At the end of data collection, the interview data appeared saturated as no new data emerged. For the study, we received an institutional review board exemption from Advarra.

We defined acceptability using the Ottawa Hospital Research Institute’s definition [19] and utilizing the understandability, clarity of information, amount of information, and suitability for decision-making themes and associated subthemes. To assess usability, we utilized the usefulness, relevance of information, value, and formatting themes and associated subthemes. This is similar to other studies that have assessed usability through the perceived usefulness, ease of use, visual design, and layout/formatting of an SDM tool [20,21].

### Data Analysis

The data analysis method was such that the interviews were transcribed, and then the transcripts were analyzed by 2 coders. The coders merged the individually coded transcripts to combine the codes and develop the basis for the analysis in NVivo. The coders used the 8 parent themes to guide the classifications of subthemes and analyzed the data from the coding of subthemes through NVivo. The coders identified the interviewees’ quotations, representative of themes and subthemes, to support the qualitative analysis. The coders coded each reference to 1 or more appropriate subthemes and the corresponding parent theme. Given that the codes were applied throughout the various sections of the PFSDM tool, the codes often overlapped, and the subthemes were not necessarily discreet instances. For example, a single section of a transcript could be coded with 2 subthemes (eg, *overall positive value* and *helps know what to expe*ct) so that the total references for the corresponding parent theme (ie, *value*) do not express that overlap. The 2 coders met regularly to compare the coded transcripts and resolve discrepancies in the application of codes. When the coders were unable to resolve discrepancies, a third member of the research team served as the arbiter. Together, 2 analysts reviewed the content and the frequency of subtheme references to come up with an overall positive and negative rating for the theme and subtheme.

## Results

### Participants

A total of 30 women with ABC participated in the validation study (Table 1). Overall, 2 Avalere Health staff conducted phone (n=25) and in-person (n=5) interviews from February to March 2019. Avalere Health staff interviewed 15 women with stage III cancer and 15 women with stage IV cancer. Participants (N=30) were all English-speaking, adult (aged ≥18 years) females with ABC (Table 1). The recruitment efforts supported a diverse mix of participants. Regarding decision-making style, no participants reported that they prefer their doctor make all of their treatment decisions without their input (Table 2).

The research team identified 8 parent themes and 35 subthemes before coding for inclusion in the final code book to comprehensively address the following 2 research questions:

- Is the PFSDM tool acceptable to patients with ABC? That is, are the components of the tool comprehensible to patients, including its length, amount of information, and overall suitability for decision making?
- Is the tool usable to patients with ABC?

When analyzing these themes, the 2 coders consistently achieved high interrater reliability, with a coding agreement above 80%. In addition, although we did not perform a stratified analysis, we purposefully recruited a diverse sample of participants (Table 1), and the participants’ responses did not differ qualitatively, based on race or education level.

**Table 1:** Participant characteristics (N=30).

Participant characteristics		Participants, n (%)
**Age (years)**		
	25-34	3 (10)
	35-44	7 (23)
	45-54	9 (30)
	55-64	9 (30)
	≥65	2 (7)
**Race/ethnicity**		
	White	15 (50)
	Black or African American	8 (27)
	Hispanic, Latino, or of Spanish origin	4 (13)
	Asian	2 (7)
	Other (eg, biracial)	1 (3)
**Location**		
	Urban	10 (33)
	Suburban	12 (40)
	Rural	8 (27)
**Education**		
	High school graduate or equivalent	4 (13)
	Some college	10 (33)
	College graduate	16 (53)
**Income (US $)**		
	Less than 25,000	6 (20)
	25,000-34,999	4 (13)
	35,000-49,999	2 (7)
	50,000-74,999	2 (7)
	75,000-99,999	4 (13)
	100,000-149,999	9 (30)
	150,000-199,999	1 (3)
	200,000 or more	2 (7)
**Insurance type**		
	Insurance through employer	17 (57)
	Medicaid	5 (17)
	Medicare	3 (10)
	Other government program (eg, Tricare)	2 (7)
	Self-purchased insurance	2 (7)
	Other	1 (3)
**Time since diagnosis**		
	0 to 6 months	5 (17)
	6 months to less than 1 year	6 (20)
	1 year to less than 3 years	10 (33)
	3 years to less than 5 years	6 (20)
	5 years or more	3 (10)
**Stage in care journey**		
	Preparing for treatment (eg, surgery or chemotherapy)	1 (3)
	Currently receiving treatment (eg, chemotherapy, radiation, hormone replacement therapy, and immunotherapy)	26 (87)
	Follow-up care (post treatment)	3 (10)

**Table 2:** Participants’ decision-making style preferences (N=30).

Decision-making style	Participants, n (%)
I prefer to make the decision about which treatment I will receive	1 (3)
I prefer to make the final decision about my treatment after seriously considering my doctor’s opinion	7 (23)
I prefer that my doctor and I share responsibility for deciding which treatment is best for me	16 (54)
I prefer that my doctor makes the final decision about which treatment will be used, but seriously considers my opinion	6 (20)
I prefer to leave all decisions regarding my treatment to my doctor	0 (0)

### Is the Preparation for Shared Decision-Making Tool Acceptable to Patients With Advanced Breast Cancer? 

#### Clarity of Information

Most interviewees reported that the information presented in the PFSDM tool was clear. They expressed that the tool had clear graphics, positive titles and instructions, and positive wording. There were a few references to items that were not clear, including terms such as *palliative care*, *prognosis*, *co-pays*, *symptom severity*, and *obtaining medications*. For example, patient 25 explained “that most people don’t even know what palliative care is.” Nevertheless, interviewees were clear on the overall message conveyed throughout the tool and felt that the information was comprehensible:

The most important thing is that the questions were easy to understand, the words weren’t necessarily clinical...

Patient 6

#### Amount of Information

All interviewees noted that the PFSDM tool did not include too much information. In fact, interviewees highlighted areas in which they would like to see additional information, including examples of strategies for reducing pain and symptoms (eg, exercise and acupuncture), important life events (eg, reunions and promotions), and living expenses (eg, housekeeping and meal preparation). Overall, the comments suggested that the tool included the right amount of content:

I think they have covered all of the bases as far as the questions are concerned. I wouldn’t remove any of them. I don’t even think I’d add any. Pretty much gotten all the areas.

Patient 3

#### Suitability for Decision Making

Almost all interviewees reported that the PFSDM tool would help patients prepare for decision making. Specifically, results indicated that the interviewees believed that the tool would help patients communicate with their providers:

I think [the tool] hit all the pertinent questions and it’s an awesome way to organize your thoughts and go into a doctor’s office with some sort of basis to stand on with questions instead of going in blindly. It gives you a direction to go.

Patient 3

Although most patients believed that the tool would prepare patients for SDM, some interviewees expressed doubt about whether their providers would participate in SDM. These participants shared experiences of not receiving straightforward answers from their providers to questions about their prognosis or a treatment’s out-of-pocket costs, their treatment’s impact on their ability to work, and potential side effects. They believed that most oncologists would not take the time to discuss the issues outlined in the tool, especially nonclinical issues. A patient reported:

I don’t feel like the doctor would actively engage me in these things. The oncologists have clinical mindsets not social.

Patient 6

Although patients might have doubts about the providers’ willingness to engage in an SDM conversation, they believe that the tool could improve their preparedness for decision making with their provider. Patients explained that the tool would help them organize their questions and stay goal-oriented before meeting with their provider. A patient stated:

[The tool] touches on a lot of things. You feel prepared for the office visit. Once you go in, your doctor asks you if you have any questions and your mind goes blank. You always miss a couple. I forgot to ask a couple questions when I saw my oncologist because I forgot to write them down. When you have this little tool here it helps a lot because you can write your notes and questions.

Patient 19

#### Understandability

Overall, patients reported understanding the PFSDM tool. The subthemes described above highlight that most interviewees could speak to the intended use of the tool and could speak clearly about the purpose of the tool. However, some interviewees did not understand which sections of the tool were educational versus those that were actionable. This confusion did not impact their overall understanding of the tool’s goals; patients still understood enough for the tool to be helpful. A patient reported:

It’s a good outline of questions to lead discussion with your doctor. It gives you an idea of what to focus on during the doctor’s appointment because you’re already so overwhelmed by so many things. It’s also a good tool to guide conversations [with your doctor] and to guide conversations with friends and family. It’s a good piece for anybody.

Patient 24

### Is the Preparation for Shared Decision-Making Tool Usable to Patients With Advanced Breast Cancer?

#### Value

Overall, interviewees felt that the PFSDM tool was valuable. Specifically, all interviewees noted that the PFSDM tool would help patients know what to expect with regard to treatment and decision making. Almost all interviewees wished they had the tool when they were first diagnosed:

I wish I would’ve had something like this when I was going through the process. There’s nothing like this unless the person who’s been diagnosed has done the research themselves. They’re too shocked to do research. You’re at the sole discretion of what the doctor tells you. So, if I had had something like this when I was first diagnosed, I think it would have opened my eyes to a lot of questions I should have asked in the beginning. I didn't. I think it’s a very good and useful tool.

Patient 8

#### Relevance of Information

All interviewees noted that the PFSDM tool or sections of the tool were relevant to patients with ABC. However, most interviewees also reported that parts of the tool were not relevant to them as individuals with ABC as they were not recently diagnosed. More broadly, patients reported that even if not all the information was relevant to them, the overall tool remained helpful:

You may not agree with everything but [the tool] could help. So much information is coming at you when you’re diagnosed, and this can help you.

Patient 17

#### Formatting

Most patients reported that the formatting of the PFSDM tool was positive. However, recurring negative comments included the need to redesign the graphics to look more like women and to rearrange certain questions in the tool to better reflect the typical flow of a conversation. For example, interviewees suggested that questions pertaining to side effects should precede those on the quality of life as it would be challenging to talk about a treatment’s impact on the quality of life before knowing the burden of potential side effects. Although some patients reported specific suggestions to improve formatting, most patients explained that the tool was well-formatted overall. Patients explained that the layout was good for note-taking and that the design was attractive. A patient reported:

I thought [the tool] had a nice flow to it, especially page 2. The questions were also laid out in a logical manner... I like that you can take notes if you would like. These are things that you can talk about and it’s focused on you, the patient. Love it.

Patient 5

#### Usefulness

All interviewees noted that the PFSDM tool was useful. The subthemes described above highlight that most interviewees could speak to the value and relevance of the tool in helping patient prepare for decision making. Those who acknowledged the less-useful portions indicated that patients are often overwhelmed within the first several weeks of diagnosis and that the questions related to prognosis and identifying biggest concerns could be especially overwhelming during this time period. Interviewees suggested that editing the instructions would improve the usefulness of a preference-specific question developed by Rocque et al:

Treatment for cancer can impact many aspects of a person’s life. We are interested in what are the most important things to you when choosing a treatment. Please choose up to three of your biggest concerns.

15

Specifically, interviewees suggested edits to the question’s instructions. They recommended that prompting patients to rank order choices instead of instructing them to select their top 3 concerns before a visit would reduce the feeling of being overwhelmed.

Nevertheless, most interviewees felt that the tool would still be useful for recently diagnosed patients. Most patients described the tool as comprehensive and helpful in outlining treatment options. A patient reported:

I think that tool should be in all breast cancer centers. Once someone is diagnosed with breast cancer, that tool should be handed to them because it gives all the options to think about. I think it’s very well done, and it should be handed out to anyone who is just finding out that they have breast cancer.

Patient 2

## Discussion

### Principal Findings

The goal of this study was to validate and update the PFSDM tool by assessing and identifying specific areas in the tool that could be modified to improve the tool’s acceptability and usability for women with ABC. Although it is important to consider men in breast cancer trials, this study focused on women as they represent approximately 99% of people diagnosed with breast cancer [22]. Therefore, future research is also needed to validate this tool for usefulness and acceptability among a wider range of patients with breast cancer beyond women, including men. In addition, we carefully considered the range of treatment-planning decisions across stage III and stage IV patients, and we wanted the tool to be inclusive of both. We found that the participants’ responses to the tool did not vary significantly by stage, indicating that both stages found the tool acceptable and usable. Overall, our study found that most participants reported that the information presented in the tool was clear and would help patients prepare for decision making. These findings are significant as the current engagement of patients with cancer in their clinical decisions is suboptimal [7,8]. As such, this tool fills this gap and helps newly diagnosed patients with ABC to (1) prepare for their treatment-planning visits, (2) structure their discussions with their care team, (3) help increase the consideration of patients’ preferences in clinical decision making, and (4) help patients and families better understand and plan for their care experiences. This validation study confirms the acceptability and usability of the PFSDM tool for women with ABC. 

### Acceptability

Our results suggest that the PFSDM tool is acceptable to patients with ABC. Overall, interviewees commented positively on the understandability, clarity, and amount of information in the tool and the tool’s suitability for decision making. Studies suggest that patient decision aids, such as this PFSDM tool, may help patients feel more knowledgeable and informed in their care decisions, therefore encouraging individuals to make treatment decisions that reflect their values [23]. Our findings support that this is true among patients with ABC engaging with the PFSDM tool as there was significant positive feedback regarding its perceived ability to help prepare patients for decision making.

Relatively equal numbers of interviewees reported that the tool did and did not include too much information. Some patients that reported that the tool did not include too much information expressed that more information could have been provided within specific sections. This finding is not surprising given the difficulty of creating a tool with the right amount of information for every patient, delivered at the optimal time.

Finally, similar to other research, although the interviewees reported that the tool would help them prepare for decision making and communicating with their providers, some interviewees expressed doubt about whether their providers would engage in an SDM conversation [24,25]. This incidental finding suggests that oncologists are not having SDM conversations. Using the PFSDM tool in the clinical setting may provide a roadmap to help patients initiate these conversations with their provider, suggesting that they have *permission* from their doctors to discuss the aspects of their care that are most important to them. Successful implementation of the PFSDM tool will require provider education and engagement on the need for SDM to drive high-quality ABC care; training on empathetic communication and how to discuss contextual or *nonclinical* topics included in the tool, such as the cost of care [26]; and wraparound patient education to signal that SDM is welcomed and is a high priority to ensure the provision of tailored, high-quality care. As this study was designed to focus on patient usability and acceptability, we collected limited input from providers at this stage. There are many steps involved in successfully implementing the PFSDM tool and multiple areas for future research, such as gathering provider input and evaluating whether the PFSDM tool affects the SDM conversation.

#### Opportunities to Improve the Preparation for Shared Decision-Making Tool for Acceptability

Participants found the PFSDM tool acceptable, and they also provided feedback about how the tool could be improved. They suggested that (1) additional information could be added to the tool (eg, examples of specific diagnostic tests or important milestones) to increase understandability, (2) the titles and instructions of each section of the tool could be improved to provide greater clarity about the intended use (ie, educational vs exercise) and audience (ie, patient vs provider), and (3) clinical terms could be better defined (eg, palliative care, prognosis, and co-pays). The representatives of patient advocacy organizations engaged in the development of this tool feel that it is important to maintain the inclusion of clinical terms, which patients will encounter in their care experience; however, patients’ desires for better definitions will be addressed. The feedback highlighted in this section will be incorporated in the next iteration of the tool to improve acceptability.

### Usability

Our results suggest that patients with ABC found the PFSDM tool to be usable. Interviewees commented positively on the usefulness, value, and formatting of the tool as well as the relevance of the information included. Patients overwhelmingly expressed that they wished they had the tool at diagnosis and that they felt the tool would have helped them know what to expect in their care.

Previous studies on patient engagement highlight that the design and the formatting of a resource are among the most important factors associated with patients’ trust in the information presented. Some of these findings also suggest that positive design features have the potential to improve the patients’ relationships with their providers [23]. Therefore, it is significant to note that the interviewees were pleased with the formatting and layout of the PFSDM tool. It is possible that this positive reaction could increase the patient’s desire to engage the tool, improving their ability to communicate their goals with their provider and leading to greater treatment satisfaction.

#### Opportunities to Improve the Preparation for Shared Decision-Making Tool for Usability

Though the perceptions of the PFSDM tool’s overall usability were high, opportunities to improve the tool and implications for its implementation emerged. Consistent with previous research highlighting that patients with advanced cancer are overwhelmed soon after diagnosis and do not know what questions to ask their doctor, some interviewees were concerned that receiving the tool shortly after diagnosis could be overwhelming. Conversely, patients desire to play a role in the clinical decision-making process and want their preferences, goals, and needs to be heard and considered [27]. Therefore, additional research should be conducted to identify the optimal time to provide the tool to patients. To address these concerns, the updates to the tool will also include the emphasis that it is not intended as a survey or mandatory paperwork for the patient to complete.

In addition, though the tool was perceived as relevant to patients with ABC overall, our results suggest that the tool may be more relevant to patients who have been recently diagnosed with ABC than those who have been living with the disease for over 1 year. For example, several interviewees noted that pages 2 and 3, which outline the broad phases of ABC care and the goals and needs to consider, respectively, would need to be updated to increase their relevance to patients already in treatment and at later stages in their care. This finding is not surprising as the tool was developed with the intention of supporting treatment planning soon after diagnosis.

As per feedback from several interviewees that it was difficult to select the 3 biggest concerns before a visit, we propose adapting the preference elicitation question in the tool [15] to provide patients with greater flexibility. We propose asking patients to rank these concerns in the order of importance, instead of just choosing 3, or ranking the concerns as high, medium, and low. The introductory language to this question should also highlight that the aim would be to cover as many concerns as possible during the visit and that the remaining concerns could be discussed during a follow-up visit. Finally, from a formatting perspective, several sections of the tool will be incorporated in the next iteration of the tool to reflect the comments provided by interviewees.

### Limitations

This study has several limitations, most of which are common in qualitative research studies. First, the PFSDM tool was not used in practice for actual decision making. Instead, patients were asked to think back on when they were first diagnosed and imagine having received this tool at that time. Second, the study results may not be generalizable outside of the population with ABC, given the small sample size. However, the results should be generalizable to other women with ABC. Third, selection bias could have been present if women who chose to participate in the study were systematically different from women who chose not to participate. Fourth, none of the participants expressed a preference for strict clinician-directed decision making (all preferred some form of an SDM process), and therefore, our results may not reflect the concerns and preferences of such patients who prefer to leave all decisions regarding their treatment to their doctor. Fifth, we did not use triangulation or respondent validation in our study, which is an area to strengthen the credibility of findings in a future study.

### Conclusions

This validation study confirms the acceptability and usability of the PFSDM tool for women with ABC. Prior studies highlighted the need to engage patients in decision making [28]. This validated tool holds promise in appropriately preparing women with ABC for their treatment panning visits and improving their engagement in decision making with their clinicians. In addition, further research is needed to test the feasibility of the tool’s implementation in the clinical setting and its ultimate impact on outcomes such as preparedness for decision making, decisional quality, and experience of care.

## Appendix

Multimedia Appendix 1 Patient survey (Web-based).

Multimedia Appendix 2 Code book.

Multimedia Appendix 3 Patient interview guide.

## Abbreviations

ABC: advanced breast cancer
PFSDM: Preparation for Shared Decision-Making
SDM: shared decision making
