# Supplementary material for: The “Preparation for Shared Decision-Making” Tool for Women With Advanced Breast Cancer: Qualitative Validation Study
Source: J Particip Med. 2019 Dec 20;11(4):e16511. doi: 10.2196/16511 (PMC7434058; doi:10.2196/16511)
Supplement: Multimedia Appendix 1 [file jopm_v11i4e16511_app1.docx]

# Multimedia Appendix 1

## Patient Survey [web-based]

Thank you for taking the time to complete this survey. We expect that this survey should take you 15-30 minutes to complete. The purpose of this survey is to receive your feedback on the Preparation for Shared Decision-Making tool, which is aimed at supporting individuals that have breast cancer and their family members. You should have received the Preparation for Shared Decision-Making tool in the mail – please have it in front of you for this survey, as some questions will ask for feedback on specific pages of the tool.

Please note that your survey answers will remain confidential. Only The Henne Group and staff from Avalere Health will have access to survey responses. Your answers will in no way affect the treatment that you receive from your doctor.

If you have any questions, please contact: Domitilla Masi, [dmasi@avalere.com](mailto:dmasi@avalere.com)

Thank you

1. **Which category below includes your age?**
2. 18-24
3. 25-34
4. 35-44
5. 45-54
6. 55-64
7. 65 or older
8. **How do you self-identify? Please check all that apply.**
9. American Indian or Alaskan Native
10. Asian
11. Black or African American
12. Hispanic, Latino, or of Spanish origin
13. Native Hawaiian or other Pacific Islander
14. White
15. Other _______________
16. **What is the highest degree or level of school you have completed?**
17. Less than high school graduate
18. High school graduate or equivalent
19. Some college
20. College graduate
21. **What is your primary language (i.e., the one you speak and/or read most of the time)?**
22. English
23. Spanish
24. Chinese (including Mandarin or Cantonese)
25. Tagalog (including Filipino)
26. Vietnamese
27. Arabic
28. French
29. Korean
30. Other_____________________
31. **What type of health insurance do you have now?**
32. Insurance through employer
33. Self-purchased insurance
34. Medicare
35. Medicaid
36. Other government program (e.g., TRICARE)
37. Not sure what type of insurance
38. I’m not insured
39. Other____________________
40. **What was your total household income before taxes during the past 12 months.**
41. Less than $25,000
42. $25,000 to $34,999
43. $35,000 to $49,999
44. $50,000 to $ 74,999
45. $75,000 to $99,999
46. $100,000 to $149,999
47. $150,000 to $199,999
48. $200,000 or more
49. **How often do you need to have someone help you when you read instructions, pamphlets, or other written material from your doctor or pharmacy?**
50. Never
51. Rarely
52. Sometimes
53. Often
54. Always
55. **Please select the stage that you believe best reflects where you are in your current diagnosis.**
56. Recently Diagnosed (no treatment yet)
57. Preparing for Treatment (e.g., surgery or chemotherapy)
58. Currently Receiving Treatment (e.g., chemotherapy, radiation, hormone replacement therapy, immunotherapy)
59. Follow-Up Care (post-treatment)
60. Other ________________
61. **How long has it been since you were first diagnosed with breast cancer?**
    1. 0 to less than 6 months
    2. 6 months to less than 1 year
    3. 1 year to less than 3 years
    4. 3 years to less than 5 years
    5. More than 5 years
62. **How many treatment decisions (e.g., about surgery, chemotherapy, hormones, radiation) have you made since your current breast cancer diagnosis?**
63. 0
64. 1 to 2
65. 3 to 4
66. More than 5
67. **How do you like making decisions about your treatment? Please choose 1 answer.**
68. I prefer to make the decision about which treatment I will receive.
69. I prefer to make the final decision about my treatment after seriously considering my doctor’s opinion.
70. I prefer that my doctor and I share responsibility for deciding which treatment is best for me.
71. I prefer that my doctor make the final decision about which treatment will be used, but seriously considers my opinion.
72. I prefer to leave all decisions regarding my treatment to my doctor.

**The following questions assess your feedback on the Preparation for Shared Decision-Making Tool.**

1. **I think …**

| 1. The amount of information is too much | Agree | Disagree |
| --- | --- | --- |
| 1. The tool will do more harm than good | Agree | Disagree |
| 1. The tool does not contain information that can help a person with breast cancer prepare for a treatment discussion visit with their doctor | Agree | Disagree |
| 1. The information is relevant to me | Agree | Disagree |
| 1. The division of the tool’s sections is presented in a clear manner | Agree | Disagree |
| 1. The “What to Expect As You Plan Your Care” (pp. 2) section is a good depiction of what a person with breast cancer can expect to experience | Agree | Disagree |
| 1. The information is understandable | Agree | Disagree |
| 1. The tool looks attractive | Agree | Disagree |
| 1. The font and font size are clear | Agree | Disagree |
| 1. The colors used are appealing | Agree | Disagree |
| 1. The instructions on how to use the tool are clear and understandable | Agree | Disagree |
| 1. I value the guidance provided in this tool | Agree | Disagree |

1. **Please answer the following questions on the acceptability of the tool.**

| - 1. How easy is the tool to use? | 1--very difficult | 2 | 3 | 4 | 5--very easy |
| --- | --- | --- | --- | --- | --- |
| - 1. How understandable is the tool? | 1—difficult to understand | 2 | 3 | 4 | 5—easy to understand |
| - 1. How valuable is the tool? | 1—not at all valuable | 2 | 3 | 4 | 5—very valuable |
| - 1. Overall, how would you rate your satisfaction with this tool? | 1—very dissatisfied | 2 | 3 | 4 | 5—very satisfied |

1. **Please rate the usefulness of the following sections of the tool, with 1 being the least useful and 5 being the most useful.**

| - 1. “What to Expect as You Plan Your Care” (pp. 2) | 1 | 2 | 3 | 4 | 5 |
| --- | --- | --- | --- | --- | --- |
| - 1. “Clarifying Your Goals & Needs” (pp. 3) | 1 | 2 | 3 | 4 | 5 |
| - 1. “Questions to Answer For Your Doctor” (pp. 4) | 1 | 2 | 3 | 4 | 5 |
| - 1. “Questions to Ask Your Doctor & Care Team” (pp. 5-9) | 1 | 2 | 3 | 4 | 5 |

1. **The length of the tool was (choose one):**
2. Too long
3. Too short
4. Just right
5. **How likely is it that you would recommend this tool to a friend or colleague diagnosed with breast cancer?**

Not at All Likely Extremely Likely

0 1 2 3 4 5 6 7 8 9 10
