# Supplementary material for: The “Preparation for Shared Decision-Making” Tool for Women With Advanced Breast Cancer: Qualitative Validation Study
Source: J Particip Med. 2019 Dec 20;11(4):e16511. doi: 10.2196/16511 (PMC7434058; doi:10.2196/16511)
Supplement: Multimedia Appendix 2 [file jopm_v11i4e16511_app2.docx]

# Multimedia Appendix 2

## Code Book

| Research Questions | Themes | Definition | Inclusion Criteria |
| --- | --- | --- | --- |
| Is the tool acceptable to patients with advanced breast cancer? i.e., are the components of the tool comprehensible to patients, including its length, amount of information, and overall suitability for decision making? | Understandability | Components of the interview related to whether patients understand how to use the tool. | Includes all comments/feedback related to whether patients understand how to use the tool. This can include comments made by note takers that are not captured in the transcript, i.e., did the patient make a comment that signifies they do not understand the aim of the tool? |
|  | Clarity of Information | Components of the interview related to the clarity of the information included in the tool. | Includes all comments/feedback related to the clarity of the: Graphics; titles; wording; and clarity among the different sections. |
|  | Amount of Information | Components of interview related to the amount (e.g., too much, too little, just right) of information included in the tool. | Includes all comments/feedback related to the amount of information present in the tool. |
|  | Suitability for decision making | Components of the interview related to whether patients feel the tool is suitable for supporting decision making | Includes all comments/feedback related to whether patients feel to tool is suitable for supporting decision making, including helping patients prepare for decision making and facilitating communication with providers. |
| Is the tool usable to patients with advanced breast cancer? | Usefulness | Components of the interview related to whether/how the tool would be used by patients. | Includes all comments/feedback related to ease of use, and which sections are more/less useful than others. |
|  | Relevance of Information | Components of the interview related to the relevance of the information included in the tool. | Includes all comments/feedback related to the tool’s specific relevance to patients with advanced breast cancer; and the patient’s stage in their care journey (e.g., recently diagnosed vs. diagnosed over 3 years ago). |
|  | Value | Components of the interview related to how valuable the tool is to patients. | Includes all comments/feedback related to the value of the tool to patients with advanced breast cancer (e.g., knowing what to expect). This can also include comments related to patients wishing they had the tool at the time of their diagnosis. |
|  | Formatting | Components of the interview related to whether the formatting is well-defined. | Includes all comments/feedback related to what the patients think of the tool’s formatting. |
