# Supplementary material for: The “Preparation for Shared Decision-Making” Tool for Women With Advanced Breast Cancer: Qualitative Validation Study
Source: J Particip Med. 2019 Dec 20;11(4):e16511. doi: 10.2196/16511 (PMC7434058; doi:10.2196/16511)
Supplement: Multimedia Appendix 3 [file jopm_v11i4e16511_app3.docx]

# Multimedia Appendix 3

## Patient Interview Guide

*Interview conducted by ______________*

*Note Taker _______________________*

*Was this interview taped? Yes__ No__*

*Name of Patient _________________________*

**Notes to interviewer**

1. *Ensure that the interviewee has been provided with a survey ahead of the call.*
2. *Read through the interviewee’s completed survey ahead of the interview.*
3. *Ask all core questions denoted with an asterisk (*).*

**Introduction**

Hello, I am ______and I work for Avalere Health, a healthcare advisory services firm based in Washington DC. Before we get started, and with the goal of keeping your answers anonymous, can you please provide me with a name – any name – that you would like to be called for this interview? Just a first name, some people prefer to use their real name and others prefer to pick a name just for this discussion.

Thank you for taking the time to speak with me today. We expect that today’s interview will take about one hour. The purpose of this interview is to get your feedback on the Preparation for Shared Decision-Making tool (the tool) aimed at supporting individuals and their family members that have breast cancer. **You should have received the tool in the mail, do you have it in front of you now?**

The tool you have in front of you was designed based on information we collected from patients with breast cancer about how they might like their needs met during treatment planning visits with their doctors. In developing this tool, we also sought input from doctors and social workers. Today, we are looking forward to getting your thoughts about this tool so that we can improve upon it to help others going through similar experiences as you.

The women who helped us develop this tool shared with us how difficult it can be to feel prepared to talk to their doctors about their treatment and get all their questions answered, especially when they are still in shock and overwhelmed by their diagnosis. The aims of this tool are to help women with breast cancer prepare for their visits with their doctors, during which they will discuss their treatments.

Our goal for today is to understand:

- Would you find this tool useful, if it were sent to you by your doctor’s office prior to your visit?
- How you feel about the language used, the way things are phrased, the amount of information that is provided, and overall the length of the tool

Please also remember that:

- - - - There are no right or wrong answers; you are the expert. We know that different people will react differently to the same information.
- Please feel free to let us know the good and the bad. The more you tell us, the more we can look for ways to improve the tool for others like you.
- We are not looking for private, or sensitive information. If we ask you any questions you don’t want to answer, feel free to opt out.

**Before we get started, do I have your permission to record today’s interview for notetaking purposes?** We will not attribute your name to any of the answers you provide. Do you have any questions before we begin the interview?

**Broad Questions:**

- Imagine that you are making an appointment for the first time with your oncologist following your diagnosis, to plan for your treatment. You call to schedule the appointment and the person you are speaking to on the phone says, “I’d like to send you some information to help you prepare for your visit with the doctor,” and “the information is intended to help you think about questions that might be useful for you and your doctor to discuss in planning for your treatment.”
- You receive this packet ahead of your visit with your doctor because your doctor wants to better understand your personal needs and preferences so those can be considered in your treatment planning and they want you to have time to think about this and possibly even discuss some of the questions with your loved ones.

*Initial Questions*

- Before we get into the “nitty gritty” of the tool, I am going to start off by asking you a general question: What is your first impression of the tool?*
  - How do you feel reading this?
  - How could you see yourself using this tool?
  - How would you describe the tool to a friend?
  - How do you feel about the design and layout? Was it attractive/appealing? Did it “look good” or put you off? Why/why not?

*Prompts (if the individual is quiet for 20-30 seconds)*

- Again, tell me what you’re thinking. Is something confusing?
  - Does it make sense? Seem organized? Why/why not?
- Is anything jumping out as particularly useful?
- Sometimes people have emotional reactions to the tool. How are you feeling?

**Detailed Questions:**

Now let’s go through each section of the tool and talk about it in more detail.

**What To Expect as You Plan Your Care (pp.2)**

- What does this page communicate to you?*
- How do you feel about the explanation for this section at the top of the page? Is it clear? What would you change or add?*
- What do you think about the graphics? Do you think this is a clear representation of what a woman with breast cancer could expect to experience in their care? Why/why not?*
- What do you think about the titles of each phase and the explanations beneath them? Are they clear?*
- Are there some words or phrasing that are not clear? Which ones?
- Thinking back on when you were first diagnosed, do you feel this section would have helped you better understand what to expect in your care?*
- What other information would you have liked? Where do you think improvements can be made?

**Clarifying Your Goals & Needs (pp.3)**

- How do you feel about the explanation for this section at the top of the page? Is it clear? What would you change or add?*
- How do you feel about the questions?*
- Are there some words or phrasing that are not clear? Which ones?*
  - Can you think of a better way to phrase them?*
- Are these questions useful to think about ahead of treatment planning? Do you think these questions would help you express your goals and needs with your doctor?*
- Can you rank the questions for us? #1 being most important?
  - Why did X question rank the lowest? What would you change about this? Would you be ok with it not being part of this tool?
- Take a moment to try to answer one of the questions on the paper (you might have already done this) – is there enough space?
- Are there improvements, other than the ones we discussed, that could be made to this page/these questions?

**Questions to Answer For Your Doctor (pp.4)**

- How do you feel about the explanation for this section at the top of the page? Is it clear? What would you change or add?*
- How do you feel about these questions?*
  - Are there questions you don’t feel belong? Why/Why not? Would you be ok with this/these not being part of this tool?*
  - Are there questions that you think belong here that you don’t see?*
  - Are there response options that are missing or should be changed?*
- Are there some words or phrasing that are not clear? Which ones?*
  - Can you think of a better way to phrase them?*
- Do you think this would be useful information to provide to your doctor? Why/why not?*
- Let’s talk about questions #4 and #5 – how do you feel about these? Are they clear? If not, how would you phrase them?
- Let’s talk about question #7 – take a minute to try to answer it for yourself. How do you feel about this question? Do you think the response options provided are comprehensive? Would you add or remove any? Which ones?
- Are there any overarching improvements, other than the ones we discussed, that could be made to this page/these questions?

**Questions to Ask Your Doctor and Care Team (pp.5-9)**

- How do you feel about the explanation for this section at the top of the page? Is it clear? What would you change or add?*
  - How might you use this section? Walk me through it.*
- How do you feel about the format?*
  - Could you see yourself taking notes on this page?*
- Let’s walk through each question and talk about whether there are some words or phrasing that are not straightforward or difficult to understand.*
  - Can you think of a better way to phrase it?*
  - Ask about the headers for the sections as well as the questions – are they understandable?*
- What do you think about the length of this section? Is this the right amount of information for you?*
- Can you see yourself asking these questions to your doctor? Do you think this section would help you identify useful questions to ask your doctor?*
- Now that we’ve walked through all the questions, which ones would you check as the most important ones to discuss with your doctor? Why?
- Which questions would you remove from this section? Why?
- Are there questions that should be here that you don’t see? What are they? Why are they important?

**Overall Discussion**

- Now that you we have walked through the entire tool together, do you think you would have wanted something like this before you started your treatment planning? Why/Why not?*
- Do you think this tool would be useful to you when preparing for a visit with your oncologist? If yes, please explain why. If no, please explain why not.*
  - Would you have liked to have used a tool like this when you were making initial treatment decisions?*
  - To what extend does this tool prepare you to make a good decision about treatment planning?*
- If you were describing this to a friend recently diagnosed with breast cancer how would you describe it?
- If you think about each section of the tool, which section is most important? Why?
  - What To Expect as You Plan Your Care (p.2)
  - Clarifying Your Goals and Needs (p.3)
  - Questions to Answer for your Doctor (p.4)
  - Questions to Ask your Doctor & Care Team (p.5-9)
- Would you recommend it to a friend to use? Why/why not?
- Are there any questions that we didn’t ask you that we should have?

**Thank you so much for your time!**
